# Occupational noise exposure and the prevalence of dyslipidemia in a cross-sectional study

**DOI:** 10.1186/s12889-021-11274-x

**Published:** 2021-06-29

**Authors:** Kun Zhang, Feng Jiang, Haibin Luo, Fangwei Liu

**Affiliations:** 1 Division of Pneumoconiosis, School of Public Health, China Medical University, No.77 Puhe Road, Shenyang North New Area, Shenyang, 110122 People’s Republic of China; 2 Health Management Center, Shenyang 242 Hospital, Shenyang, People’s Republic of China

**Keywords:** Occupational noise exposure, Dyslipidemia, Lasso-logistic regression, Restricted cubic spline

## Abstract

**Background:**

Occupational noise exposure was related to cardiovascular disease, of which dyslipidemia was an important inducement. This study investigated the relationship between occupational noise exposure and dyslipidemia.

**Methods:**

Four hundred ninety-two occupational noise-exposed workers and 664 non-exposed workers were recruited to conduct environmental noise tests and personal occupational physical examinations. A lasso-logistic regression model was used to estimate the relative risk of dyslipidemia. A restricted cubic spline was used to estimate the association between noise exposure years and dyslipidemia after adjusting for potential confounding factors.

**Results:**

A crude association was observed between the occupational noise exposure (75–85 dB(A)) and dyslipidemia. After adjusting for confounding factors, there was a non-linear relationship between noise exposure years and dyslipidemia (P for non-linearity =0.01). Workers exposed to 75–85 dB(A) for 11 to 24.5 years had a higher risk of dyslipidemia than non-exposed workers.

**Conclusions:**

A positive and non-linear exposure-response relationship was found in workers exposed to 75–85 dB(A) whose exposure years were between 11 and 24.5. Workers had the highest risk of dyslipidemia when exposed for 13.5 years.

**Supplementary Information:**

The online version contains supplementary material available at 10.1186/s12889-021-11274-x.

## Background

As an environmental and occupational pollutant, the detrimental effect of noise has attracted more and more attention [1, 2]. Epidemiological studies have reported increased mortality and incidence of cardiovascular diseases after chronic occupational noise exposure [3–6]. Noise, a psychosocial stressor, could lead to the increase of blood lipids, blood pressure, and blood glucose, which were the pathological basis of cardiovascular disease [7]. The possible biological mechanism was that noise impaired the hormones and metabolic regulation by activating the sympathetic nerve and endocrine system [8, 9]. Therefore, studying the effects of noise on blood lipids, blood pressure and blood glucose was vital for the prevention of cardiovascular disease.

At present, a large amount of evidences show that noise exposure could cause an increase in blood pressure and blood glucose [10–13]. However, there were relatively few studies on the relationship between noise and blood lipids. Some researchers have observed that there were increased cholesterol and triglyceride when workers were exposed to occupational noise above 80 dB(A) [14, 15]. Nevertheless, dyslipidemia has not been confirmed by others [4, 16, 17]. A study manifested no association between occupational noise exposure and dyslipidemia, although adjusted for 11 confounding factors such as sex, age, and body mass index (BMI) [18]. In addition, the effect of noise on blood lipids needed to be accumulated over a long period of time, but few studies explored the relationship between noise exposure years and blood lipids.

Nowadays, The least absolute shrinkage and selection operator (lasso)-logistic regression and restricted cubic spline (RCS) have been used to analyze the relationship between disease and risk factors [19, 20]. Lasso-logistic regression was a combination of lasso regression and logistic regression. When the regression model had plentiful covariates, the stepwise selection approach was not optimal, while lasso regression had many desirable properties [21, 22]. Lasso regression has been proven to effectively eliminate multicollinearity among confounding factors [23]. Therefore it could screen out the factors which had a direct impact on the dependent variable among these confounding factors. The restricted cubic spline could detect the possible nonlinear relationship between the dependent variable and the main independent variable through a continuous curve [24]. While the two methods have not been applied to screening and analyzing the relationship between occupational noise exposure and dyslipidemia.

The purpose of this study was to use a more advantageous model to screen confounding variables and investigate the exposure-response relationship between occupational noise exposure and the risk of dyslipidemia.

## Methods

### Study population

Two aviation manufacturing plants were randomly selected from all aviation manufacturing companies in Shenyang. In the two plants, all workers working in noise exposure workplaces were selected as the exposure group. The non-exposed group was included the workers who had no specific noise exposure in the workplace in the same plant and met the hygienic standard for noise in industrial enterprises (GB 3096–2008). A total of 1513 workers were included.

The inclusion criteria were workers without a family history of deafness, dyslipidemia, or other cardiovascular diseases; history of head trauma, blast exposure, hearing system disease, major liver disease, and ototoxic drug; and missing data. According to the Occupational Health Standard of the People’s Republic of China: Measurement of Noise in the Workplace (GBZ/T 189.8–2007) (China, 2007), the Chinese workplace noise exposure limit was 85 dB(A), and this study aimed to explore the impact of noise below it on blood lipids. What’s more, some studies which also considered the impact of lower-lever noise exposure on non-auditory systems chose 75 dB(A) as the cut-off value [18, 25, 26]. Therefore, the workers with occupational noise exposure had a duration of more than 1 year and the intensity of noise exposure was 75–85 dB (A) (LEX,8 h).

Each participant performed an occupational health examination in 2018, which included a history and physical examination (Additional file 1, was developed for this study). In the exposed group, noise exposure years were recorded, which was the working years of workers who were still working in the noise exposure workshop and have not been transferred by the physical examination. Professional physicians determined the height, weight, heart rate, electrocardiogram, blood pressure, blood glucose, blood lipids, fatty liver, and hearing impairment. Body mass index (BMI) was calculated by dividing body weight (kg) by the square of height (m).

Finally, 492 occupational noise-exposed participants (exposed group) and 664 non-noise-exposed participants (non-exposed group) were included in this cross-sectional study.

### Definition of dyslipidemia

Blood tests were performed to assess triglyceride (TG), serum total cholesterol (TC), high-density lipoprotein cholesterol (HDL-C), and low-density lipoprotein cholesterol (LDL-C). The judgment of dyslipidemia referred to the standards of the “Guidelines for the Prevention and Treatment of Dyslipidemia in Adults in China (2016 Revised Edition)”: (1) TC ≥6.2 mmol/L; (2) HDL-C < 1.0 mmol/L; (3) LDL-C ≥ 4.1 mmol/L; (4) TG ≥ 2.3 mmol/L. Dyslipidemia was defined as the presence of one or more of these components.

### Assessment of noise exposure

Noise intensity in the workplace was determined by a noise statistical analyzer (HS6288E). According to the occupational health standard in China (GBZ/T 189.8–2007), noise exposure was evaluated with equivalent continuous dB(A)-weighted sound pressure levels (LEX,8 h). Based on the occupational noise exposure assessment, the intensity of noise in the workplace was 75–85 dB(A) (LEX,8 h).

### Assessment of pure-tone audiometry

The degree of hearing impairment was identified by pure-tone audiometry. Based on the National Occupational Health Standard of the People’s Republic of China, an audiometer (AD28) was used to measure the air conduction hearing thresholds at the speech frequencies(500,1000,2000 Hz) and the high frequencies(3000,4000,6000 Hz). The hearing thresholds for these frequencies 26 dB(A) or higher in any ear were defined as the increased hearing threshold.

### Statistical analysis

SAS (university edition) was used to analyze descriptive statistics between the noise exposure group and the non-noise exposure group. The Shapiro-Wilk test was used to assess the normality of continuous variables. Because continuous variables distributed abnormally, summary values were median (P25, P75). Categorical variables were expressed as frequencies (%). Sorted all variables in layers, the χ^2^ test was used to compare the differences between groups.

Tibshirani proposed a penalty regression method—lasso in 1996, which was characterized by variable selection and complexity adjustment while fitting a generalized linear model [23]. Regardless of the nature of the dependent variable, lasso regression could be used for modeling.

The basic idea of lasso regression was that each regression coefficient was compressed by a penalty function, and even some regression coefficients became 0, so that a simplified model with fewer variables could be obtained. The logistic regression model established by the lasso method for variable selection was the lasso-logistic regression model.

This study discussed the effect of noise exposure on blood lipids, which was a binary model problem. According to the conditions of dyslipidemia prevalence, all participants were divided into non-diseased and diseased, where 0 meant non-diseased and 1 meant diseased. Since the conditions of dyslipidemia prevalence were independent of each other, let (X_i_, Y_i_) be the sample value of the dyslipidemia condition of n workers. X_i_ was the observed value of p different attributes of the i-th worker. Y_i_ was the dependent variable, and Y_i_ ∈{0, 1}. The probability of a worker suffering from dyslipidemia was assumed π_i_ = p(Y_i_ = 1|X_i_). β was the logistic regression coefficient, and λp(β) was the penalty term (1).

1 $$ \lambda p\left(\beta \right)=\lambda \sum \limits_{j=1}^p\left|{\beta}_j\right| $$

Then the estimated coefficient $\hat{\beta}$ in the lasso-logistic regression model could be written in the form of the formula (2).

2 $$ \hat{\beta}=\mathit{\arg}\mathit{\min}\left[-\sum \left\{{Y}_i\mathit{\ln}\left({\pi}_i\right)+\left(1-{Y}_i\right)\mathit{\ln}\left(1-{\pi}_i\right)\right\}\right]+\lambda p\left(\beta \right) $$

The first half of the formula (2) expressed the degree of model fitting, and the second half expressed the punishment for variables. λ (λ ≥ 0) was used as a penalty parameter to determine the degree of compression of model coefficients. The penalty function was to punish the absolute value of the regression coefficients, requiring the sum of the absolute values of all regression coefficients to be less than or equal to the penalty parameter λ. According to the requirements of penalty conditions, $\sum \limits_{j=1}^p\left|{\beta}_j\right|\le \lambda$. As λ increased, the coefficients of each variable were gradually compressed. When λ → ∞, all coefficients were compressed to zero.

In the lasso model, the size of the penalty parameter λ was directly related to the complexity and convergence of the final model. Therefore, choosing a reasonable and scientific parameter tuning method was of great significance to the final effect of the lasso model. The simplest and most widely used method to select the appropriate value of λ was the cross-validation method [27]. In this method, the samples were divided into n sub-samples, and the n groups of sub-samples were cross-validated n times to obtain n times the model fitting situation, from which the optimal λ was selected.

Therefore, the Lasso regression model was used to screen the risk factors of dyslipidemia and indicated the degree of importance of all factors. The cross-validation method was used to calculate λ value with the smallest error, which corresponded to the screened risk factors. A binary logistic regression model was used to assess the association between noise exposure status and dyslipidemia, with adjusting for the other screened factors. The results were presented as odds ratio (OR) with their 95% confidence intervals (CI). A restricted cubic spline was used to flexibly model the association of duration of noise exposure (year) and dyslipidemia prevalence. In the spline model, other screened factors were adjusted. The non-linearity assumption was tested by using a likelihood ratio test.

In addition, a *p*-value of < 0.05 was used to define statistical significance in all analyses. Apart from descriptive statistics, other statistical analyses were conducted in R (version 3.6.2).

## Results

In total, 1513 workers were included in the study. The baseline characteristics of participants in both the exposed group and the non-exposed group were shown in Table 1, of whom most were focused on the age of 30–39. The population of the study included more males than females in both groups. Significant differences were identified between the exposed group and the non-exposed group in sex, BMI, PTA, hypertension, fatter liver, and diabetes (all P’s < 0.05). The median of noise exposure years was 13(8, 19) years, with a noise intensity value of 78.90(78.10, 82.10) dB(A).

**Table 1:** Demographic Characteristics of Employees by Workplace Noise Exposure

Variables	Noise-exposed group			control group			*P* value^a^
	No.	%	M (QL, QU)	No.	%	M (QL, QU)	
**Age**			35(29, 39)			34(30, 39)	0.300
< 30	136	27.64		160	24.10		
30–39	251	51.02		376	56.63		
40–49	67	13.62		80	12.05		
50–59	38	7.72		48	7.23		
**Sex**							< 0.001
Male	461	93.70		471	70.93		
Female	31	6.30		193	29.07		
**Heart Rate**			77(71, 85)			79(72, 86)	0.259
< 60	12	2.44		8	1.20		
60–100	466	94.72		634	95.48		
> 100	14	2.85		22	3.31		
**BMI**			25.29(22.66, 27.96)			23.73(21.22, 26.20)	< 0.001
< 18.5	12	2.44		28	4.22		
18.5–23.9	161	32.72		325	48.95		
24–27.9	200	40.65		242	36.45		
≥ 28	119	24.19		69	10.39		
**ECG**							0.254
Normal	362	73.58		508	76.51		
Abnormal	130	26.42		156	23.49		
**PTA**							< 0.001
Normal	465	94.51		664	100.00		
Abnormal	27	5.49		0	0.00		
**Hypertension**							< 0.001
Yes	210	42.68		188	28.31		
No	282	57.32		476	71.69		
**Fatty liver**							< 0.001
Yes	279	56.71		243	36.60		
No	213	43.29		421	63.40		
**Diabetes**							0.008
Yes	22	4.47		12	1.81		
No	470	95.53		652	98.19		
**Noise exposure years (years)**	13 (8, 19)						
**Noise intensity (dB(A))**	78.90(78.10, 82.10)						

*Abbreviations*: *BMI* body mass index, *ECG* electrocardiogram, *PTA* pure-tone audiometry

^a^ χ^2^ test for the difference (*P* < 0.05) between the 2 groups

The median levels of TG, TC, HDL, and LDL and the odds ratio of dyslipidemia between the two groups were presented in Table 2. Compared with the non-exposed group, the exposed group had significantly higher levels in TG, TC, and LDL (*P* < 0.001). Workers exposed to noise had a higher odds ratio of dyslipidemia (unadjusted odds ratio = 1.81,95%CI:1.40,2.35).

**Table 2:** Rest of blood lipids and odds ratio of Dyslipidemia by Noise Group, Shenyang, Liaoning,2018

Noise Group	Median (QL; QU) Blood lipids (mmol/L)				No. of dyslipidemsia	Risk of Dyslipidemia (Unadjusted)		
	TG	TC	HDL	LDL	Cases	OR	95%CI	*P* Value ^b^
Control	1.13(0.73;1.81)	4.74(4.21;5.41)	1.43(1.22;1.71)	2.60(2.20;3.10)	153	1.00	Referent	
Noise-exposed	1.44(0.99;2.43)	4.89(4.25;5.56)	1.30(1.18;1.46)	2.82(2.33;3.30)	173	1.81	(1.40,2.35)	< 0.001
*P* Value ^a^	< 0.001	< 0.001	< 0.001	< 0.001				

*Abbreviation*: *TG* triglyceride, *TC* serum total cholesterol, *HDL* high-density lipoprotein, *LDL* low-density lipoprotein, *OR* odds ratio, *CI* confidence interval

^a^ Wilcoxon two-sample test for the difference between the two groups

^b^ Simple logistic regression model

**Fig. 1:**
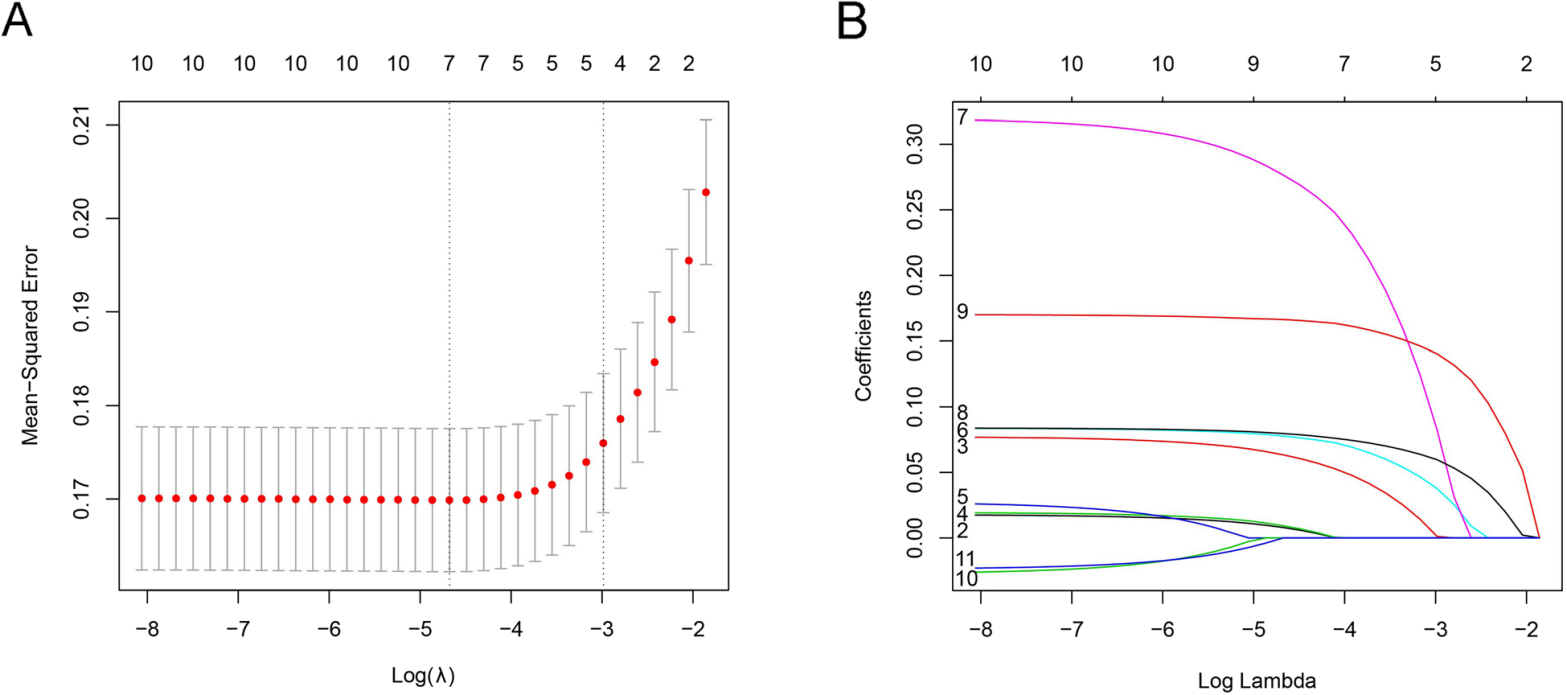
The screening of variable. **a** The trend between lambda(λ) and the number of variables. A cross-validation method was used. **b** The relationship between lambda(λ) and lasso coefficients.2, Age;3, Sex;4, Noise exposure;5, PTA;6, Hypertension;7, Diabetes;8, BMI;9, Fatty liver;10, Heart rate;11, EGC

After baseline description, age, sex, heart rate, BMI, ECG, PTA, hypertension, fatty liver, and diabetes were incorporated into the Lasso regression model, which was used to screen variables. Lambda(λ) value was shown by the cross-validation method in Fig. 1(a). The range between the two dashed lines indicated the range of positive and negative standard deviations of the lambda value. Within the range, the deviation of the regression model fluctuated slightly. Thus, the lambda value with the smallest mean-squared error in the range was chosen, which included 7 variables. The coefficient of each risk factor was compressed as the lambda value became larger (Fig. 1(b)). The earlier the variable was compressed, the less important it was. Ultimately the top seven risk factors, fatter liver, BMI, hypertension, diabetes, sex, noise exposure, and age, were retained.

**Fig. 2:**
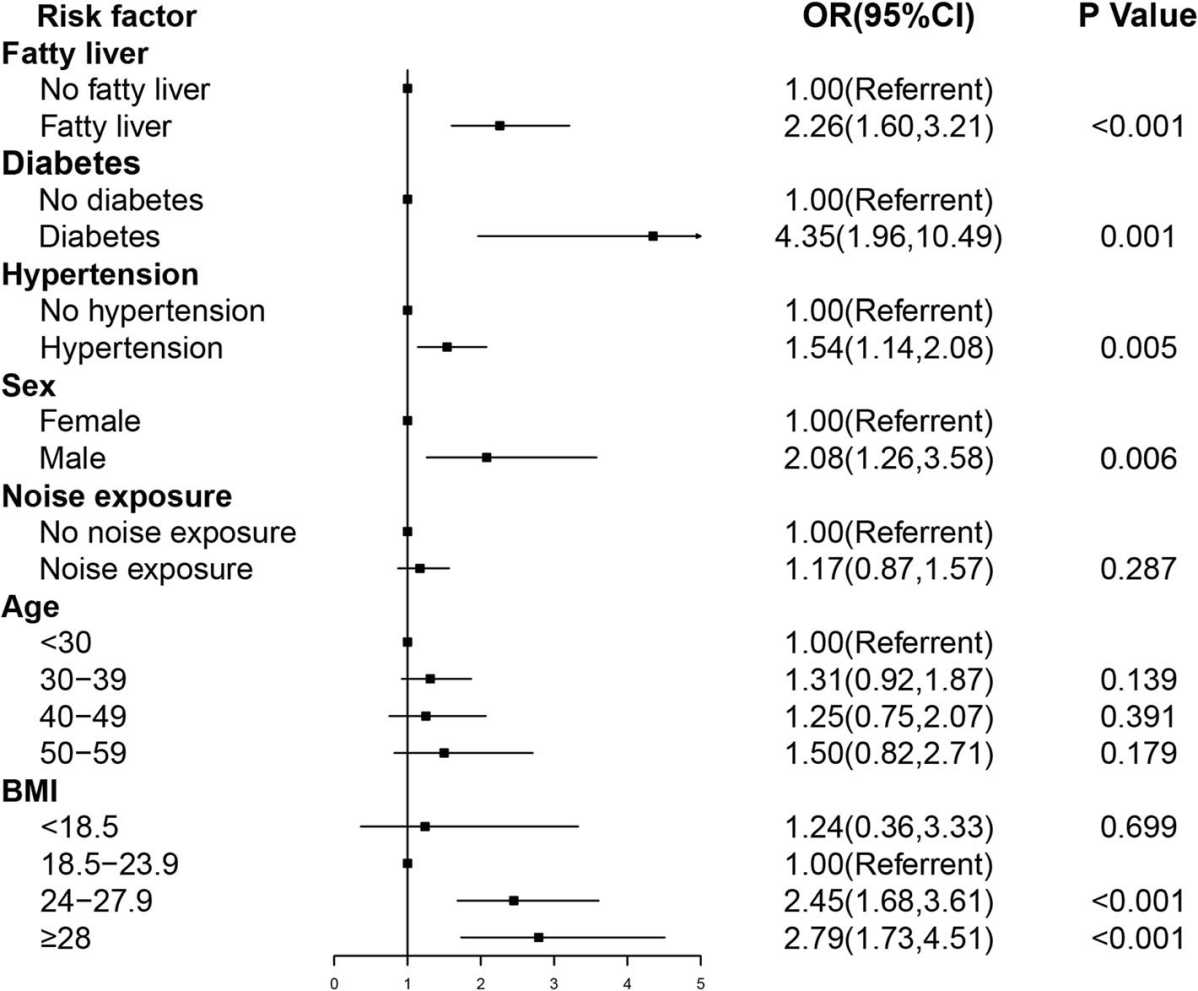
Forest plot for performance on the odds ratio of prevalent dyslipidemia by exposing occupational noise**.** A logistic regression model was used. OR, odds ratio; BMI: body mass index; CI, confidence interval

Thus, the 7 selected variables were included for logistic regression analysis. The association between noise exposure and prevalence of dyslipidemia was shown in Fig. 2. After adjusting for fatty liver, diabetes, hypertension, sex, age, and BMI, no significant difference was found in the relationship between occupational noise exposure and prevalence of dyslipidemia (P’s > 0.05). Workers with a BMI of 24–27.9 had a higher relative risk of dyslipidemia than workers with a BMI of 18.5–23.9, and the strongest association was observed at the BMI of ≥28. Participants with fatty liver, diabetes, hypertension, and male had an increased probability of having dyslipidemia, respectively.

**Fig. 3:**
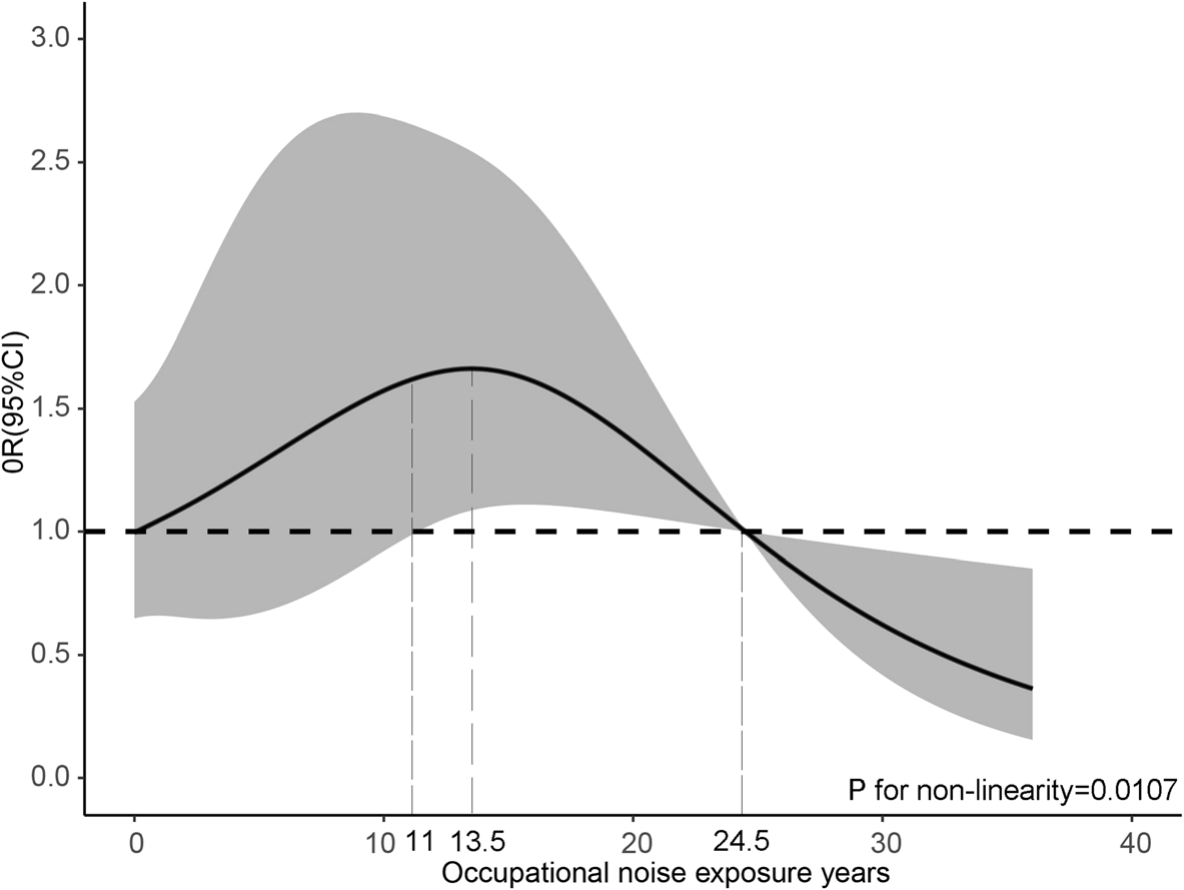
Association (odds ratio) between occupational noise exposure years and dyslipidemia prevalence among 1156 participants. A restricted cubic spline with 4 knots was modeled. Analysis was adjusted for fatty liver, diabetes, hypertension, sex, age, and BMI.OR, odds ratio; CI, confidence interval

Further, the exposure-response relationship between the continuity of noise exposure and dyslipidemia is worthy of further research. Therefore, the noise exposure years were introduced to explore the effect of long-term exposure on dyslipidemia. A restricted cubic spline was used to observe the relation of noise exposure years and prevalence of dyslipidemia in Fig. 3. After adjusting for fatty liver, diabetes, hypertension, sex, age, and BMI, the risk of prevalent dyslipidemia was not significantly correlated with noise exposure years until around 11 years and then increased during 11–13.5 years and decreased above 13.5 years (P for non-linearity =0.01).

## Discussion

### Summary

The study explored the correlation between occupational noise exposure and the prevalence of dyslipidemia by modeling. First, well-established extraneous risk factors of blood lipid levels were accounted for and screened. Interestingly, the crude association between occupational noise exposure and dyslipidemia vanished after adjusting for the screened confounders. Second, we explored the long-term changes of chronic noise exposure to dyslipidemia. Our findings suggested there was a non-linear relationship between noise exposure years and dyslipidemia among workers in aviation manufacturing plants. Workers exposed to occupational noise (75–85 dB(A)) for 11–24.5 years had a higher prevalence of dyslipidemia than those without noise exposure.

### Lasso-logistic regression---screening and linear analysis of risk factors of dyslipidemia

Epidemiological evidence has demonstrated that long-term exposure to noise increased the risk of cardiovascular disease [2, 28]. Occupational noise-induced dyslipidemia might be an important intermediate link in subsequent major cardiovascular events. But, the actual relationship between occupational noise exposure and dyslipidemia was still under investigation. Their association was observed in young men(≤44 y) after adjusting for a limited set of extraneous risk factors [14]. Another study also showed a significantly different level of TG between noise-exposed workers and non-exposed workers after adjustment for some confounders [29]. On the contrary, some researchers demonstrated that there was no association between noise exposure level and blood lipid levels [4, 16, 17]. Therefore, novel perspectives and appropriate methods were needed to explore the real relationship between occupational noise and dyslipidemia.

In this study, ten recognized risk variables, noise exposure, age, sex, heart rate, BMI, ECG, PTA, hypertension, fatty liver, and diabetes were included to explore the association between occupational noise and dyslipidemia. The relationships among these risk factors were complicated and had a certain correlation. Existing studies about the impact of occupational noise exposure on dyslipidemia were mostly using stepwise logistic regression to screen variables. However, the results obtained by using this method were inherently discrete and unstable so that it was difficult to screen the most meaningful variables [30, 31]. Our study used Lasso regression to screen out seven variables: noise exposure, age, sex, hypertension, fatty liver, diabetes, and BMI. Because lasso regression had the advantages of strong predictive ability and high stability. What’s more, it retained a few significant variables and eliminated the influence of multicollinearity by reducing the insignificant coefficient to 0 [23]. After that, the screened variables were included in the logistic regression model analysis. The processes of screening and analyzing variables were called lasso-logistic regression. Our results have shown that there was no association between occupational noise exposure and dyslipidemia when other confounding factors were considered, which were consistent with others [18]. The reason might be that the noise intensity in the study was weaker than the national exposure limit of 85 dB(A) (LEX,8 h), and low-level noise might have less impact on human physiology. A study found a similar result that when the noise intensity was below 85 dB(A) (LEX,8 h), no significant difference was found in the risk of hypertension between noise-exposed and non-exposed groups [32].

### The exposed-response relationship between noise exposure years and dyslipidemia

Existing theories believed that the changes in blood pressure were unstable in the early stage of noise exposure. And long-term exposure to high-intensity noise could lead to a continuous increase in blood pressure [33, 34]. Similarly, the early effect of noise exposure on blood lipids was also not significant [35]. Moreover, some researches focused on the relationship between the intensity of noise and dyslipidemia, hypertension, and other diseases [26, 36, 37]. Our study included the continuous index of noise exposure years in order to observe the effect of long-term noise exposure on blood lipids. Because of the unknown relationship between noise exposure years and dyslipidemia, blindly using segmented regression could unify the internal effects of each segment. Importantly, it was difficult to find the maximum and minimum points in this method, which were obvious shortcomings in observing the exposed-response relationship. In order to avoid the influence of model selection and impertinent grouping of noise exposure years, a restricted cubic spline combined with a logistic linear regression model was used to explore the relationship between noise exposure years and dyslipidemia. By this method, the effect of a small change in the independent variable was visualized with a continuous curve [24].

This study has shown that there was a non-linear exposed-response relationship between noise exposure years and dyslipidemia after adjusting for confounding factors. With the increase of noise exposure years, the risk of dyslipidemia presented a trend of rising first then falling. When the noise exposure years were between 11 and 13.5, the risk of dyslipidemia gradually increased, which indicated that the risk of dyslipidemia increased with the accumulation of noise exposure time. The risk of disease gradually decreased after 13.5 years of exposure. The reason might be that due to the physical deterioration or the occurrence of chronic diseases, some of the workers exposed to noise were transferred from their original positions, which gradually reduced the risk of dyslipidemia. Another study shown younger workers exposed to noise (> 80 dB(A)) demonstrated a significant increase in TG, TC, and TC/HDL ratio, which confirmed our suspicions [14].

### Limitations

To analyze the relationship between noise exposure and dyslipidemia more comprehensively, some factors can be considered in the future. Workers who have been exposed to noise by being transferred from the noise exposure position were not included, because they might have more complicated confounders that affected their blood lipids during the physical examination, such as the nature of new jobs and the resulting change in living and eating habits. What’s more, the risk factors of dyslipidemia are complex, thereby some factors are still worth being explored, such as protective measures, living and eating habits, and genetic factors. In addition, participants’ exposure to noise intensity outside of the workplace might not be measured. Furthermore, this study is a cross-sectional study, so causality could not be determined.

### Prediction and prevention of dyslipidemia

In this study, the prediction model formed by lasso-logistic regression and restricted cubic spline had good prediction performance. The obtained exposure-response model could be used as a tool for predicting dyslipidemia for workers exposed to noise. The model showed that when the noise intensity was 75-85 dB(A) and the noise exposure years were 11–24.5, the prevalence of dyslipidemia increased.

On the one hand, according to the occupational health standard in China (GBZ/T 189.8–2007), the limit value for the prevention of auditory effect of occupational exposure to noise in China is 85 dB(A). Although this limit was often used to discuss non-auditory effects [3], there was still a lack of limit values for the non-auditory effect of occupational noise exposure. Few studies used 75 dB(A) as the cut-off level. A study that only included workers exposed to noise found that when workers exposed to less than 75 dB(A) were used as a reference, workers exposed to 75-85 dB(A) were associated with hypertension [25]. Further, compared with non-noise-exposed workers in our study, with using more appropriate methods for modeling, workers exposed to 75-85 dB(A) of noise for a long time would still cause non-auditory effects such as dyslipidemia. Therefore, even if the noise was below the current limit of 85 dB(A), the risk of non-auditory diseases would still increase. This finding may provide a theoretical basis for establishing Chinese limit values for the prevention of non-auditory effects of occupational noise exposure.

On the other hand, enterprises are supposed to strengthen regular monitoring and early intervention of risk factors, especially for workers with more than noise exposure years at 11. It is vital to pay attention to the implementation of noise prevention and control measures and strengthen the personal protection of noise-exposed workers. Equally important, it is recommended to focus on providing nutrition intervention and health education to workers suffering from overweight and obesity, hypertension, diabetes, and fatty liver. Moreover, workers who have developed dyslipidemia can transfer from exposed positions in time to avoid aggravation or development of cardiovascular disease.

## Conclusions

In summary, occupational noise exposure may be associated with an increased prevalence of dyslipidemia. Our study found a positive and non-linear exposure-response relationship was found in workers exposed to 75–85 dB(A) whose noise exposure years were between 11 and 24.5. Workers had the highest risk of dyslipidemia when exposed for 13.5 years.

## Supplementary Information

**Additional file 1.** Occupational Health Examination Form (history examination section). This questionnaire collected the basic information, work position, occupational history, past disease history, family disease history, occupational disease history, menstrual history, reproductive history, smoking and drinking history, and symptom inquiry during examination.

## Data Availability

The datasets used and analysed during the current study are available from the corresponding author on reasonable request.

## Abbreviations

BMI: Body mass index
Lasso: Least absolute shrinkage and selection operator
RCS: Restricted cubic spline
TG: Triglyceride
TC: Serum total Cholesterol
HDL-C: High-density lipoprotein cholesterol
LDL-C: Low-density lipoprotein cholesterol
OR: Odds ratio
CI: Confidence intervals
ECG: Electrocardiogram
PTA: Pure-tone audiometry
