# Supplementary material for: Occupational noise exposure and the prevalence of dyslipidemia in a cross-sectional study
Source: BMC Public Health. 2021 Jun 29;21:1258. doi: 10.1186/s12889-021-11274-x (PMC8243570; doi:10.1186/s12889-021-11274-x)
Supplement: Supplementary file 1 — Additional file 1. Occupational Health Examination Form (history examination section). This questionnaire collected the basic information, work position, occupational history, past disease history, family disease history, occupational disease history, menstrual history, reproductive history, smoking and drinking history, and symptom inquiry during examination. [file 12889_2021_11274_MOESM1_ESM.doc]

Name:

Company Name:

Company Telephone:

Employee Number:

Serial Number:

Date of Filling:

Category: Pre-job ( )

On-the-job ( )

Off-job ( )

Occupational Health Examination Form

(history examination section)

Name: Gender:

ID number: Marital status:

Type of work: working years:

Type and name of occupational hazard exposure:

Signature:

Date:

1. Occupational history (filled out by the inspected person)

| Start and end time | Company name | Workshop | Type of work | Harmful factors | Protective measures |
| --- | --- | --- | --- | --- | --- |
|  |  |  |  |  |  |
|  |  |  |  |  |  |
|  |  |  |  |  |  |
|  |  |  |  |  |  |
|  |  |  |  |  |  |
|  |  |  |  |  |  |
|  |  |  |  |  |  |
|  |  |  |  |  |  |
|  |  |  |  |  |  |

1. Past disease history

1. Family disease history

IV. Acute and chronic occupational diseases history

Disease name: Date of diagnosis:

Hospital of diagnosis: Cured or not:

V. Menstrual history

Age of menophania: Age of menopause:

Days of menstruation: menstrual cycle:

1. Reproductive history:

How many children do you have?

Have you ever had an abortion? If yes, how many times?

Have you ever had a premature birth? If yes, how many times?

Have you ever had a stillbirth? If yes, how many times?

Have you ever had an abnormal fetus? If yes, how many times?

VII. Smoking and drinking history:

Smoking: never ; occasional ; a lot .

How many cigarettes per day ; for how many years: .

Drinking: never ; occasional ; a lot .

How many milliliters you drink per day ; for how many years:

VIII. Symptoms (if the inspected person suffers from the following symptoms, it is indicated by "+". If not, it is indicated by "-".)

| Item | date | Item | date |
| --- | --- | --- | --- |
| 1.Headache |  | 35.Panting |  |
| 2.Dizziness |  | 36.Chest distress |  |
| 3.Dazzling |  | 37.Pectoralgia |  |
| 4.Insomnia |  | 38.Cough |  |
| 5.Drowsy |  | 39.Expectoration |  |
| 6.Dreaminess |  | 40.Hemoptysis |  |
| 7.Memory weaken |  | 41.Asthma |  |
| 8.Temperament |  | 42.Palpitation |  |
| 9.Fatigue |  | 43.Precordial discomfort |  |
| 10.Low fever |  | 44.Anorexia |  |
| 11.Night sweats |  | 45.Wasting |  |
| 12.Hyperhidrosis |  | 46.Nausea |  |
| 13.Body aches |  | 47.Vomiting |  |
| 14.Hyposexuality |  | 48.Bloating |  |
| 15.Blurred vision |  | 49.Bellyache |  |
| 16.Decreased vision |  | 50.Liver pain |  |
| 17.Ophthalmalgia |  | 51.Diarrhea |  |
| 18.Photophobia |  | 52.Astriction |  |
| 19.Lacrimation |  | 53.Frequent urination |  |
| 20.Hyposmia |  | 54.Urgent micturition |  |
| 21.Nasal cavity dryness |  | 55.Hematuresis |  |
| 22.Stuffy nose |  | 56.Subcutaneous hemorrhage |  |
| 23.Nosebleed |  | 57.Pruritus |  |
| 24.Runny nose |  | 58.Erythra |  |
| 25.Tinnitus |  | 59.Puffiness |  |
| 26.Deafness |  | 60.Alopecia |  |
| 27.Thirst |  | 61.Arthronalgia |  |
| 28.Salivation |  | 62.Acroanesthesia |  |
| 29.Toothache |  | 63.Inflexible action |  |
| 30.Loose teeth |  | 64.Abnormal menstruation |  |
| 31.Bleeding from brushing teeth |  | 65. |  |
| 32.Oral malodor |  | 66. |  |
| 33.Oral ulcers |  | 67. |  |
| 34.Sore throat |  | Doctor's signature |  |
